# Polymer Hydrogels and Frontal Polymerization: A Winning Coupling

**DOI:** 10.3390/polym15214242

**Published:** 2023-10-27

**Authors:** Alberto Mariani, Giulio Malucelli

**Affiliations:** 1 Department of Chemical, Physical, Mathematical and Natural Sciences, University of Sassari, Via Vienna 2, 07100 Sassari, Italy; mariani@uniss.it; 2 Consorzio Interuniversitario per la Scienza e Tecnologia dei Materiali, INSTM, Via Giusti 9, 50121 Firenze, Italy; 3 Department of Applied Science and Technology, Politecnico di Torino, Viale Teresa Michel 5, 15121 Alessandria, Italy

**Keywords:** hydrogels, frontal polymerization, structure–property relationships, applications

## Abstract

Polymer hydrogels are 3D networks consisting of hydrophilic crosslinked macromolecular chains, allowing them to swell and retain water. Since their invention in the 1960s, they have become an outstanding pillar in the design, development, and application of engineered polymer systems suitable for biomedical and pharmaceutical applications (such as drug or cell delivery, the regeneration of hard and soft tissues, wound healing, and bleeding prevention, among others). Despite several well-established synthetic routes for developing polymer hydrogels based on batch polymerization techniques, about fifteen years ago, researchers started to look for alternative methods involving simpler reaction paths, shorter reaction times, and lower energy consumption. In this context, frontal polymerization (FP) has undoubtedly become an alternative and efficient reaction model that allows for the conversion of monomers into polymers via a localized and propagating reaction—by means of exploiting the formation and propagation of a “hot” polymerization front—able to self-sustain and propagate throughout the monomeric mixture. Therefore, the present work aims to summarize the main research outcomes achieved during the last few years concerning the design, preparation, and application of FP-derived polymeric hydrogels, demonstrating the feasibility of this technique for the obtainment of functional 3D networks and providing the reader with some perspectives for the forthcoming years.

## 1. Introduction

In the last 30 years, the biomedical and pharmaceutical sectors have greatly benefited from the design and application of polymeric hydrogels, i.e., three-dimensional hydrophilic networks made of crosslinked macromolecular chains, exhibiting high swelling in water and water retention, external stimuli responsiveness, and tunable mechanical features [1,2,3,4,5,6,7]. Because of these properties, polymeric hydrogels have found several applications in different sectors, ranging from the “traditional” biomedical field to their quite recent use for flexible aqueous energy storage devices (as hydrogel electrolytes) [8,9].

**Figure 1:**
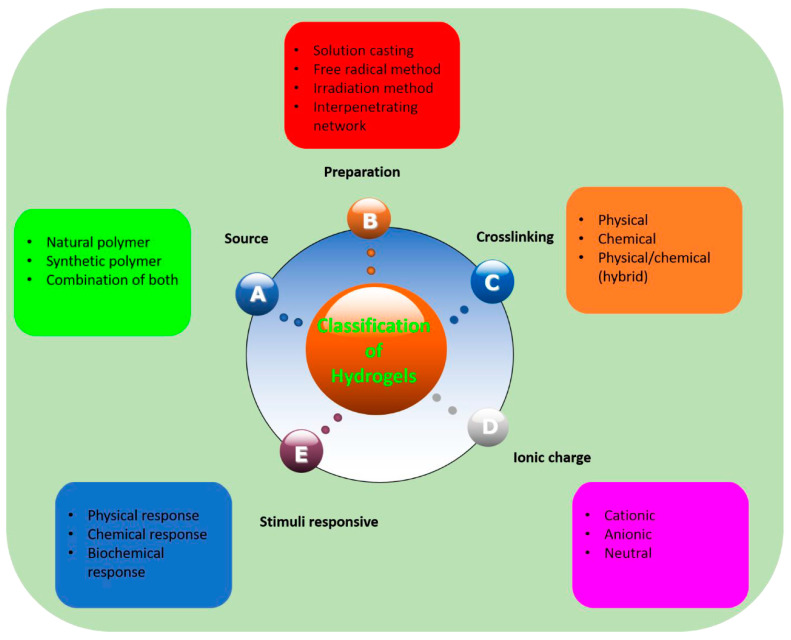
Schematic classification of polymeric hydrogels. Reprinted from ref. [4] under Creative Commons CC-BY 4.0 License.

Figure 1 elucidates how polymeric hydrogels can be classified, according to different parameters, namely, the source of the hydrogel (i.e., natural or/and synthetic materials), the preparation method (i.e., through free radical polymerization reactions, gamma or UV irradiation, interpenetrating network formation, solution casting, or simple mixing), the type of crosslinking (i.e., chemical, physical, or hybrid), the possible ionic charge (anionic, cationic, or neutral systems), and even the type of stimuli responsiveness (i.e., biochemical-, chemical-, or physical-responsive hydrogels).

As reported in the scientific literature, all the aforementioned synthetic strategies for polymer hydrogels are well-established and optimized, although they are usually time consuming, encompass quite complex reaction routes, and require quite a high amount of energy. In this context, the frontal polymerization (FP) technique [10,11,12] may represent an alternative strategy that can limit the drawbacks associated with the synthesis of polymeric hydrogels. At the same time, it gives rise to the formation of tailored 3D networks fulfilling the requirements of the envisaged applications. FP takes advantage of the formation of a hot polymerization front that, if the exothermicity of the polymerization reaction is sufficient, is capable of self-sustaining and propagating throughout a monomeric medium, hence rapidly converting monomers into polymers. The corresponding experiments are most often carried out in test tubes containing monomer mixtures rising 5 to 10 cm high. Apart from some monomeric systems that frontally polymerize in only a few seconds [13], usually, FP mixtures require longer times (i.e., from tenths of seconds to a few minutes), which are, however, much shorter than their counterparts polymerized in batches. Further, the thermal and mechanical properties of the latter are often lower with respect to those of frontally polymerized systems [14,15,16,17]. 

Indeed, because of the higher temperature reached by the polymerization front as compared with the temperature typically used for the same synthesis performed using the classical protocol, conversion is often higher. In turn, this results in a more regular polymer network and porosity, which accounts for the higher *T*_g_ and improved mechanical behavior (in particular, tensile and compression moduli) observed in this regard [18,19].

**Figure 2:**
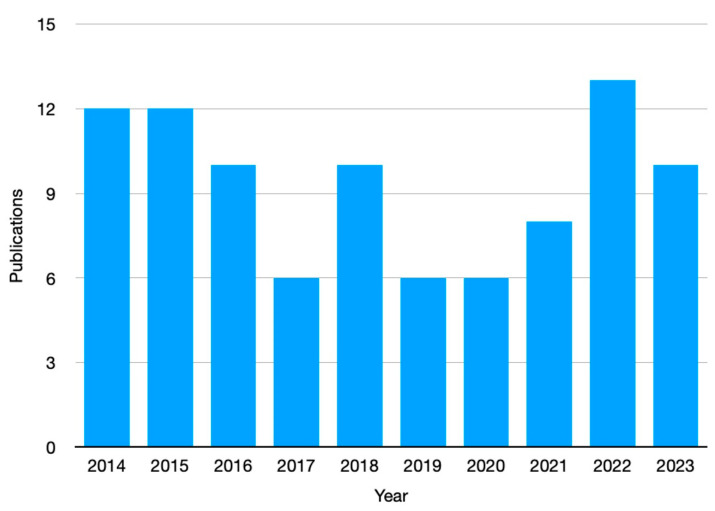
Number of publications (from 2014 to 2023), in peer-reviewed journals, dealing with “hydrogels AND frontal polymerization” (data collected from the Web of Science^TM^ database, www.webofscience.com, accessed on 25 June 2023).

The interest in the use of frontal polymerization for preparing advanced hydrogels is evidenced by the number of peer-reviewed articles published in the scientific literature during the last ten years (Figure 2).

Therefore, in the present review, we will expound upon the basics of the general characteristics of frontal polymerization. Then, some specific features of the FP technique applied to the synthesis of hydrogels will be provided. Eventually, the most recent outcomes on this topic will be presented and discussed, including with respect to their possible applications. It should be highlighted that a comparison between FP and batch techniques was reported in almost all the pioneering studies on frontal polymerization only. Later, FP was considered a suitable and reliable method that does not need to be proven to be better or worse than the traditional ones. As a consequence, we anticipate that the possible differences arising from the use of the FP and batch techniques will be described only when mentioned in the recent published papers, i.e., the object of the present review.

## 2. Fundamentals of the Frontal Polymerization Technique

The following paragraphs deal with some general principles and aspects typical of frontal polymerization, which can be useful for readers who are not familiar with the technique. For more details, we encourage such readers to refer to the reviews published by Suslick et al. [12], Khan and Pojman [20], Li et al. [11], and Pojman [21].

### 2.1. Definitions

**Figure 3:**
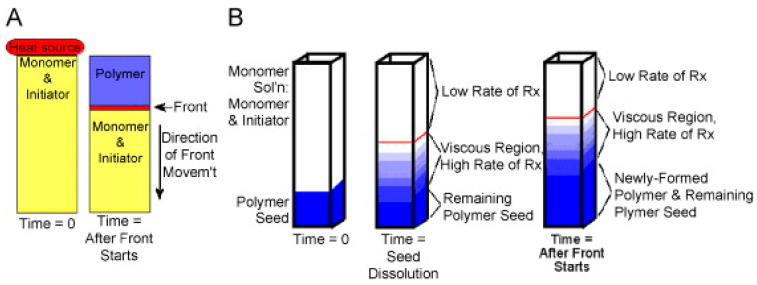
(**A**) Thermal frontal polymerization and (**B**) isothermal frontal polymerization. (Reprinted with permission from Ref. [22]. Copyright Elsevier, 2008).

In the state of the art, there are two main types of frontal polymerization: isothermal and thermal FP (Figure 3).

In isothermal frontal polymerization [22,23], the gel effect is exploited. This is a phenomenon observed mainly in the polymerization of some acrylic monomers and occurs when the viscosity of a monomer mixture becomes high enough. Under these conditions, diffusion becomes unlikely. More specifically, a slowing down of the termination rate is observed in radical polymerizations due to the decreased diffusion efficiency of the growing macromolecules. In addition to the increase in the average length of macromolecular chains, an increase in the concentration of radicals and, consequently, in the polymerization rate is also observed.

In practical terms, upon comparing the polymerization rate of the same monomer mixture in two different media, one with low viscosity and the other with high viscosity, it can be gleaned that the polymerization rate is higher under the latter conditions. On this basis, the insertion of preformed polymer seeds capable of swelling when placed inside a low-viscosity monomer mixture results in the polymerization rate of the monomer within the polymer being greater than that outside, resulting in an increase in the size of the seed. At this point, it is easy to imagine why the conversion of a monomer to a polymer occurs mainly at the interface between the high-viscosity and low-viscosity phases. Therefore, it can be observed that polymerization takes place due to the formation of a propagating front (Figure 3B). From a practical point of view, to prevent the amount of heat that develops from being excessive, there is a tendency for this type of reaction to occur at a low and constant temperature (even below 10 °C), hence the name isothermal frontal polymerization.

However, when we discuss frontal polymerization, we tend to refer to its variant that exploits the exothermicity of the reaction; in its most complete definition, this variant is called thermal frontal polymerization. However, in this review, although the adjective thermal is omitted, in speaking of frontal polymerization or FP, we will refer only to this second type of macromolecular synthesis.

In this case, for FP to occur, it is necessary for the polymerization reaction to be sufficiently exothermic. More specifically, let us initially consider a polymerization reaction promoted by increasing temperature and conducted according to the classical technique, namely, taking into consideration the simplest system, i.e., the bulk polymerization of a liquid monomer. The most common approach, or at least the most efficient, is to thermostat a reactor to a certain temperature, the choice of which is determined by kinetic, thermodynamic, energy, safety, and cost considerations. Ideally, the goal is to initiate a reaction in the shortest possible time while operating safely and ensuring the creation of a polymer product that meets the required characteristics.

This implies that while thermostatting a reactor to a relatively high temperature leads to decreased process times, it also induces higher energy costs, more pollution, and a greater likelihood of reaction runaway with decreased safety and loss of control over the final material properties. Conversely, the use of low process temperatures solves safety and energy-saving issues but involves long times with consequent negative effects on efficiency.

Frontal polymerization is an interesting alternative that is intended to solve at least some of the problems listed above. In its most common protocol, it is carried out in an elongated, generally cylindrical reactor (most often a test tube), which is not insulated (but can be if required by the corresponding process parameters) and not thermostated. Through an external energy source (usually heat or light), polymerization is induced in a localized area of the reaction mixture. Since the reaction is exothermic (this requirement is essential), locally, there is a sufficient temperature rise for the reaction to proceed spontaneously without requiring a further input of external energy.

Given that the hot zone that is polymerizing is in contact with the monomer mixture that has not reacted yet, it can be guessed that enough heat exchange occurs at the interface to cause a local initiation of the reaction. The result is the growth of the hot polymerized phase at the expense of the cold monomeric phase. This growth is nothing more than a hot front of polymerization that ideally propagates until the whole monomer is converted into a polymer (Figure 3A).

Some aspects must be taken into account, which we will briefly summarize below. For further discussion, we refer the reader to other publications [12,23].

**Figure 4:**
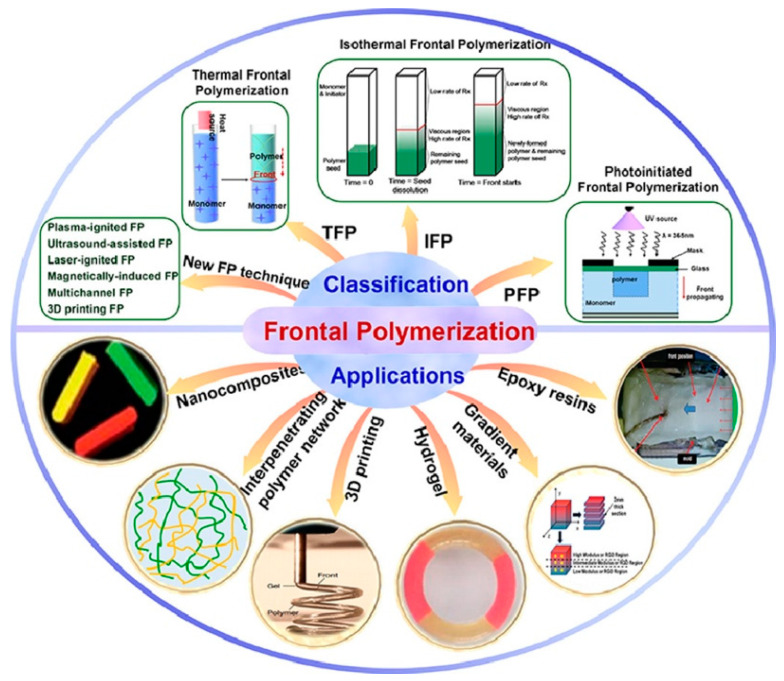
Some types and applications of frontal polymerization. Reprinted with permission from Ref. [11]. Copyright Elsevier, 2022.

Some types and applications of frontal polymerization are reported in Figure 4.

### 2.2. Reactor Characteristics

As mentioned above, in the majority of the cases reported in the literature, frontal polymerization reactions are conducted in cylindrical reactors (test tubes). However, this condition is not necessary, but it is convenient on a laboratory scale. Instead, what matters most is the surface area. In fact, as it increases, the amount of heat dissipated outward by the curing system also increases up to a critical limit. At this point, the remaining heat becomes insufficient for enabling the front’s self-sustainment.

Thus, even tubular reactors that are too thin may not be suitable for FP to take place. Similar considerations apply to films or other materials in the form of thin layers. If the surface area exposed to the outside is large relative to the total volume of the curing mass, FP may not occur. However, films prepared using FP have been reported [12].

### 2.3. Direction of Front Propagation and Stirring

**Figure 5:**
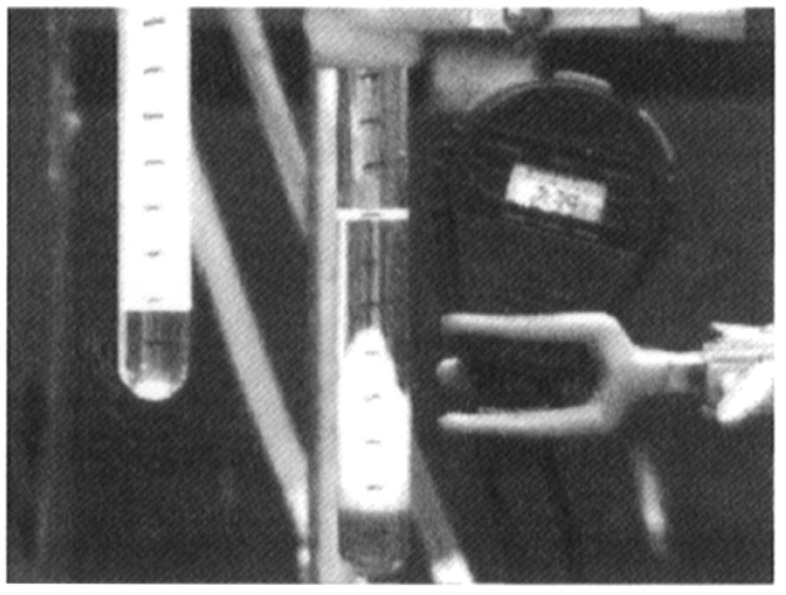
Examples of descending (**left**) and ascending (**right**) fronts. (Reprinted with permission from Ref. [25]. Copyright American Chemical Society, 1997.

In general, it is preferable to induce FP by triggering it from above so that the front propagates downward. Given the upward direction of convective motions, this might seem counterintuitive, and upward propagation might seem to be the favored direction. In fact, to avoid the quenching of the polymerization front, one generally operates under conditions such that any motion of the polymerizing mixture is avoided, at least in the direction perpendicular to the propagation of the front. In the case of parallel motions, particularly those having the same direction as the front, on the other hand, the relevant risk is that the front will lose its condition of stability and assume elongated shapes. This results in an increase in the surface area in contact with the outside or with the cold monomer, causing the front to eventually destroy itself (Figure 5) [24,25].

### 2.4. Fingering

**Figure 6:**
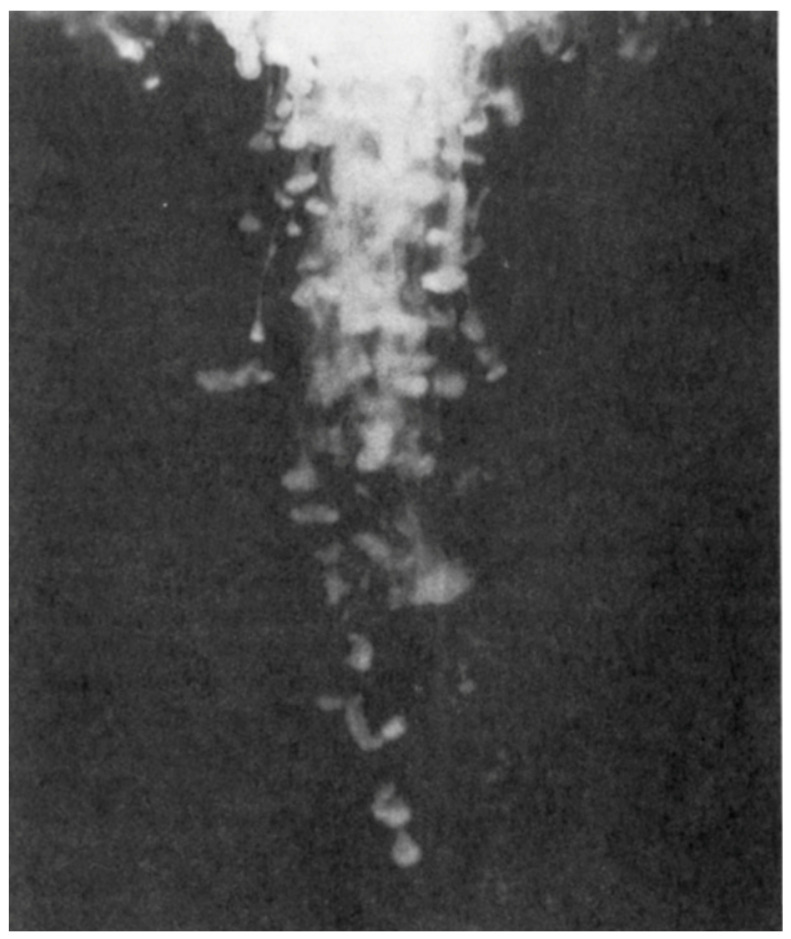
Fingering phenomena during the frontal polymerization of methacrylic acid. Reprinted with permission from Ref. [26]. Copyright American Chemical Society, 1992.

This is a phenomenon related to the previous aspects and is another possible reason for front quenching that can occur in the case of descending fronts. The hot polymer that begins forming at the expense of the underlying monomer is easily soluble in this monomer; alternatively, it could have a density such that it drips and infiltrates into the monomer [26]. If the refraction index of the monomer is sufficiently different from that of the polymer, such an infiltration visually manifests as “fingers” creeping into the underlying phase, hence the name fingering (Figure 6). Since this is a phenomenon that occurs in the same direction as the front, it leads to the front’s quenching, often associated with the initiation of polymerization in the areas of the monomer that have come into contact with the hot fingers, thus causing bulk polymerization.

### 2.5. Monomers

In the most common cases, the monomers used are liquids. However, there are examples where front polymerization also occurs on solids (e.g., acrylamide and dicyclopentadiene) [12,23]. Obviously, in principle, solid monomers are unfavorable since their polymerization occurs through their melting, which, being an endothermic process, subtracts heat from the hot front, thus making its propagation more critical. On the other hand, solid monomers have the advantage of not inducing fingering phenomena. Therefore, the choice of monomers suitable for descending frontal polymerization is generally limited to those giving rise to thermosets or polymers that have little or no solubility in a polymerization mixture. Generally, this condition is achieved through the addition of multifunctional comonomers, which give rise to crosslinking.

Another determinant characteristic is the boiling temperature of the monomer. Indeed, given the temperatures reached in polymerization fronts (typically between 120 and 180 °C [12], although FP reactions occurring outside this range have been widely reported) [24], there is a possibility that the monomer will boil when heated by the approaching front. In this case, bubbles will form and remain entrapped in the final polymeric material. However, since boiling is an endothermic phenomenon, its occurrence implies the removal of heat from the system and the possible quenching of the front. Of course, it is possible to perform this procedure under pressure; however, in laboratory experiments, mostly aimed at demonstrating the feasibility of FP under the simplest possible conditions, this possibility is generally ignored.

### 2.6. Mechanisms, Reaction Kinetics, Initiators, and Catalysts

Among the requirements for FP to be successfully carried out, pot life plays a key role. In fact, the monomer mixture must be stable at relatively low temperatures (generally, at room temperature) but must react quickly at the front temperature. While, on the one hand, this ensures that the monomer does not spontaneously react in areas far from the front, which would result in undesirable bulk polymerization, on the other hand, it guarantees that the heat of polymerization is released as quickly as possible, thus avoiding the monomer’s excessive dissipation that would prevent the front from self-sustaining and propagating.

Most of the monomers successfully used in FP are acrylic or methacrylic, which are polymerized through radical chain polymerization [12,27]. However, step-growth kinetics or different mechanisms have also been reported (e.g., polyurethanes [28,29,30] and epoxy resins [31,32]).

In particular, some systems have been obtained through catalytic processes [12] (e.g., polydicyclopentadiene) or through cationic polymerization, including radical-induced cationic frontal polymerization, among others [33,34,35].

Another important aspect is the concentration of the initiator or catalyst used. In fact, as already pointed out, in order to exploit as much heat of polymerization as possible, it is important that this heat is developed quickly so that the system does not have time to dissipate it outward. This is reflected in the concentrations of the initiator or catalyst, which are often found to be higher than those typically employed in similar reactions conducted according to traditional protocols. However, as they increase, two phenomena can be observed: a decrease in pot life, resulting in a tendency for the system to polymerize spontaneously in bulk, and the generation of an excessive amount of heat such that the achieved temperature of the front turns out to be too high to guarantee a final material having the desired characteristics and to ensure a safe operation. On the other hand, excessively low concentrations of an initiator or catalyst entail a slowing down of the reaction kinetics, which results in heat amounts that, at a given time, are too low to initiate monomer polymerization, thus generating fronts that are unable to self-sustain or do not form at all.

Therefore, there is a general tendency to define and operate within appropriate initiator or catalyst concentration ranges.

Another aspect of great importance lies in the decomposition products of the initiators. For example, azo-bis-isobutyrronitrile is known to decompose via releasing nitrogen, and benzoylperoxide can release CO_2_. Therefore, to avoid the formation of bubbles that remain within the final material, efforts have been made to use gas-free initiators, which are often purposefully synthesized [27,36].

Nevertheless, it is still sometimes necessary to add appropriate activators or inhibitors [34,37] to the system. They aim to promote the reaction under particularly unfavorable conditions or to increase the pot life of the system, respectively.

It is also worth pointing out that, especially in the case of acrylic or methacrylic monomers, these can often be used in their original states, without requiring their purification through the removal of the inhibitors added by the supplier. In fact, the temperatures reached by the polymerization fronts are generally high enough to make their presence almost kinetically irrelevant.

### 2.7. Solvents

In many cases, FP is conducted in the presence of solvents, which further complicates its management. In fact, in addition to the well-known considerations about the advantages and disadvantages of polymerization in a solution compared to that conducted in the absence of solvents, performing FP in the presence of solvents further complicates the polymerization process, limiting the potential use of this technique. Specifically, one of the first requirements relates to their boiling temperature, which—to avoid bubbles and heat absorption due to evaporation—must be higher than that of the polymerization front. This limits the use of the most common solvents, including water; however, several frontal polymerizations have been successfully performed in this medium, [12] including those carried out for the obtainment of hydrogels such as the ones that are the subject of this review. Furthermore, among others, FPs conducted in ether, chloroform, acetone, benzene, etc., are, in fact, impracticable unless one operates under pressure (see above).

Heat capacity is another issue related to the presence of the solvent (actually, it is referred to as such for all components of the mixture). In systems in which the exothermicity of the reaction is relatively low, the presence of a solvent—which absorbs some of the heat released—makes FP even more critical, with consequent effects on other process parameters, including the concentration of the initiator or catalyst, the geometry of the reactor, and its degree of insulation. On the other hand, the presence of a solvent can be useful in increasing the pot life of the system and decreasing the front temperature (if excessively high).

Thus, the influence of solvent power is related to the previous considerations. In general, the tendency is to limit the use of solvents as much as possible. Thus, a good solvent is preferred to avoid excessive dilution and consequent heat dissipation.

### 2.8. Viscosity

In order to stabilize the fronts, especially the descending ones but also the horizontal ones, one can choose to increase the viscosity of the monomer mixture. This way, phenomena such as fingering turn out to be prevented to some extent as the easy diffusion of the polymeric phase into the monomeric one is disfavored. For this purpose, it is possible to opt for particularly viscous solvents (e.g., glycerol) or for the addition of appropriate viscosifiers (e.g., polymers, fumed silica, etc.) [38].

For particular applications (e.g., in putties) [33], the high viscosity resulting from the addition of various inert components to the monomer is of paramount importance to ensure the propagation of fronts in any direction.

### 2.9. Composites

One of the most interesting applications of FP involves the obtainment of composite and nanocomposite materials. Regarding the former, in which the amount of filler can be even greater than that of the monomer, some of the foreseeable consequences include a decrease in temperature and front velocity as the additive content increases. For these systems, in which the monomer is in fact diluted in a heat-absorbing medium, a limiting composition must be identified, above which the amount of filler is too high to ensure a front’s self-sustainment.

Obviously, given that the filler concentration is typically no more than a few percentage units, in nanocomposites, this phenomenon is less likely. Furthermore, nanocomposites obtained via frontal polymerization can benefit from an additional advantage over analogs prepared using traditional techniques as the result of the much greater rate at which the monomer is converted into a polymer. For these systems, this factor is not insignificant, as examples have been reported in which nanoparticles that would otherwise tend to re-aggregate in monomer-to-polymer conversion actually remain dispersed as their diffusion turns out to be particularly slow compared to the rate of polymerization. As an example, we cite graphene during the frontal polymerization of *N*-isopropylacrylamide [39,40].

## 3. Hydrogels Obtained via Frontal Polymerization

Obtaining hydrogels via frontal polymerization represents one of the possible applications of FP and, provided the general requirements outlined above and the more specific ones illustrated below are met, is a particularly convenient method because of its simplicity and short time of execution. The latter aspect is particularly useful not only in the study of frontal polymerization per se but also in providing rapid indications about the characteristics of new materials whose obtainment will subsequently be scaled up to industrial production, with considerable savings in time and cost.

### 3.1. Monomers

The main characteristic of a hydrogel is, of course, its compatibility with water, the concentration of which—in the case of non-crosslinked systems—can be high enough to dissolve the material [4].

In general, the hydrophilicity of a polymeric hydrogel depends on that of the starting monomers, which must therefore be highly polar and, if possible, ionizable. In the next paragraph, a series of polymeric hydrogels will be presented, and their characteristics will be discussed in view of their current or potential applications.

It should be specified that frontal polymerization cannot be used to obtain the whole class of hydrogels that can be synthesized via the classical route.

This depends, first of all, on the possible polymerization reactions involved in the process but also on the intrinsic features of the various individual monomers.

In fact, many hydrogels are obtainable through reactions that are not compatible with the characteristics required by FP, most importantly the exothermicity of the reaction. In particular, polycondensation reactions are very unlikely to be carried out frontally because the condensation coproduct’s formation is an endothermic process that subtracts some of the heat made available by the macromolecular chain growth reaction.

Moreover, focusing, for example, on the radical polymerization of acrylic or methacrylic monomers, which are probably the most widely used in FP, a problem related to the molar mass of the monomer to be polymerized immediately appears. As a general rule, in fact, a small monomer consisting of a C-C double bond and a few other atoms is more likely to be frontally polymerized than a larger one. The reason for this lies in the number of atoms not involved in the polymerization process, which, if present in a high number, tend to absorb an excessive amount of the heat released due to the exothermicity of the reaction, thus preventing the front from self-sustaining [41]. The obvious consequence related to the low molecular mass of the monomer is thus the limited number of possible compounds and isomers.

As will be shown later by discussing the reviewed publications, the easiest way to increase the number of available systems lies in copolymerization and functionalization [42,43], the latter being generally employed to impart particular properties to the final material. Relative to copolymerization, as will be seen, the tendency is to use a reasonably small number of monomers (e.g., acrylamide, acrylic, itaconic acid, *N*-isopropylacrylamide, 2-hydroxyethylacrylate, etc.), having good hydrophilicity and capable of frontally polymerizing, and copolymerize them with other compounds. However, precisely because of the considerations about front self-sustainment, the volume fraction of the latter cannot be very high.

### 3.2. Solvents

In the most favorable cases, it is possible to carry out a reaction in water. Of course, this implies that all the components of the reaction mixture are soluble in this medium and that the amount of heat absorbed by this solvent (known to have high heat capacity and heat of evaporation) does not prevent the self-sustainment of the polymerization front.

However, as hydrophilic as monomers may be, they might not be water-soluble, a problem that becomes even more dramatic in the case of copolymers or functionalized systems. Thus, it is often necessary to use other solvent media, usually those that are polar. Examples of these include dimethylsulfoxide, *N*-methylpyrrolidone, glycerol, ionic liquids, and deep eutectic solvents [11].

### 3.3. Initiators

In the case of operating in water, the preferred initiators generally belong to the persulfate class [27,36]. Alternatively, if the solvent is different or not present, more compounds can be used. Furthermore, the presence of any bubbles within the hydrogel may not be a problem or may even be a desired feature. If this is the case, the number of available initiators is greater than that in the frontal polymerization of other materials.

### 3.4. Front Temperature and Material Characteristics

A large proportion of commonly used polymeric hydrogels have been used in applications at near room temperature, including the physiological one. In many cases, these are materials that undergo physical, if not chemical, changes at relatively low temperatures.

For example, poly(*N*-isopropylacrylamide) exhibits a lower critical solution temperature (LCST) in water at about 30–32 °C [39]. This characteristic, which determines a drastic decrease in hydrophilicity, could be a problem, e.g., in the presence of third components. Indeed, the fact that this material is swollen at room temperature (and thus below the LCST) while being shrunken at the face temperature could affect the desired characteristics of the final copolymer or composite material.

## 4. Applications of Frontally Polymerized Hydrogels

Frontally polymerized hydrogels have been finding interesting applications in various advanced sectors. This paragraph will summarize the main outcomes achieved in the last eight years, which clearly demonstrate the importance and feasibility of exploiting the frontal polymerization method for the rapid and low-energy-consuming synthesis of hydrogels for advanced applications.

### 4.1. Biomedical Applications

Nuvoli et al. [44] succeeded in synthesizing double-responsive (i.e., to pH and temperature) hydrogels based on poly(*N*-vinylcaprolactam-co-itaconic acid). The itaconic acid content in the comonomer mixture allowed for the possibility of performing the frontal copolymerization reaction in a quantitative yield and was responsible for the stimuli-responsive characteristics of the obtained hydrogels. In particular, as this comonomer bears allylic hydrogens inhibiting radical polymerization, frontal copolymerization occurred only when the itaconic acid content was kept below 10 mol %. Further, the hydrogels containing 1 mol % of the comonomer exhibited a lower critical solution temperature at around 30 °C, like that of poly(*N*-isopropylacrylamide), the most well-known thermoresponsive hydrogel. In addition, all the synthesized hydrogels were pH-responsive, and this behavior was strictly correlated with the presence of itaconic acid. More specifically, below pH = 7, the comonomer units induced increased hydrophobicity and hence lowered swelling. Conversely, beyond pH = 7, an increase in both hydrophilicity and the swelling ratio was observed due to itaconic acid units bearing carboxylate groups.

**Figure 7:**
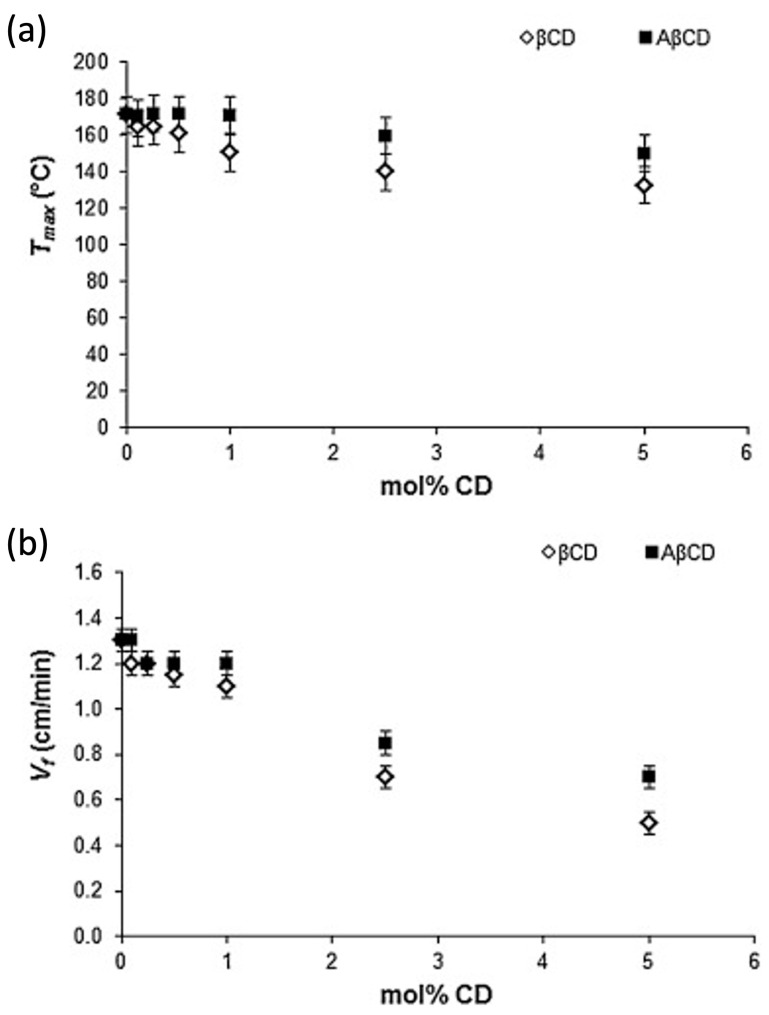
Front temperatures *T*_max_ (**a**) and front velocities *V*_f_ (**b**) as a function of the molar amount of β-cyclodextrin (βCD, white symbols) and acryloyl-β-cyclodextrin (AβCD, black symbols). Reprinted with permission from [45]. Copyright Elsevier, 2016.

In a further research effort, Nuvoli and co-workers [45] exploited frontal polymerization for preparing two types of poly(2-hydroxyethylacrylate)-based hydrogels containing β-cyclodextrins that were either directly dispersed into the acrylic polymer or grafted (as acryloyl-β-cyclodextrin) onto poly(2-hydroxyethylacrylate) chains. The presence of 0.5 mol % of cyclodextrins, irrespective of their type, accounted for the maximum swelling ratio (about +300% with respect to neat poly(2-hydroxyethylacrylate)). Concerning the frontal polymerization reaction, both front temperatures (*T*_max_) and front velocities (V_f_) were found to decrease when increasing the cyclodextrin loading (Figure 7). This finding was ascribed to the fact that the cyclodextrins behaved as an inert material, favoring heat dissipation. It is worth noticing that heat dissipation was reduced when acryloyl-β-cyclodextrins were utilized; the latter also promoted a significant increase in the glass transition temperature of the dry hydrogels compared to that of neat poly(2-hydroxyethylacrylate) (43 °C vs. 20 °C, respectively) because of an increased crosslinking effect induced by the functionalized β-cyclodextrins. 

**Figure 8:**
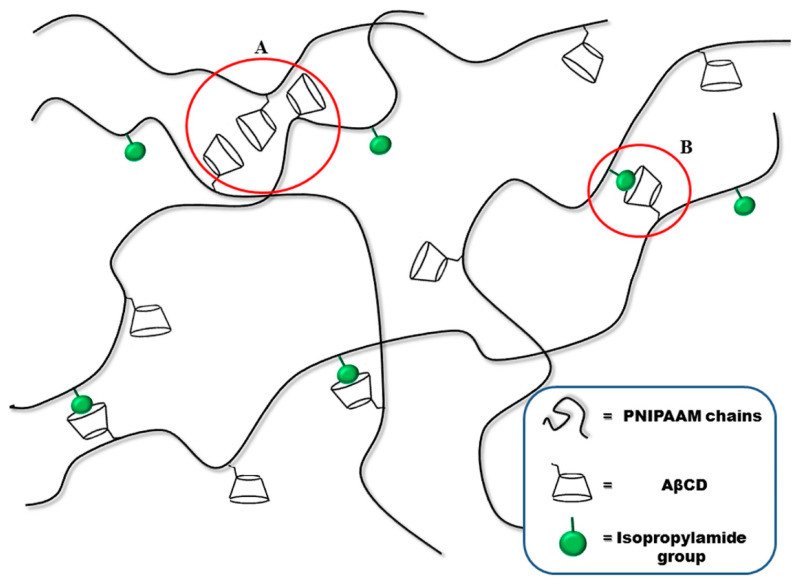
Supramolecular crosslinking in poly(N-isopropylacrylamide) hydrogels due to both mutual β-cyclodextrin interaction (**A**) and host–guest (i.e., inclusion complex) formation (**B**) with isopropylamide groups. Legend: PNIPAAM = poly(*N*-isopropylacrylamide); AβCD = acryloyl-β-cyclodextrin. Reprinted with permission from [46]. Copyright Elsevier, 2017.

The same group [46] further demonstrated the successful synthesis of poly(*N*-isopropylacrylamide)-grafted acryloyl-β-cyclodextrin hydrogels via FP. Notably, it was possible to obtain crosslinked hydrogel structures by exploiting supramolecular interactions only, i.e., without employing any chemical crosslinkers, as schematized in Figure 8. The overall behavior of the supramolecularly crosslinked hydrogels was compared with that of covalently crosslinked counterparts, using *N*,*N*′-methylene-bis-acrylamide as a crosslinker. The presence of AβCD in both types of hydrogels did not affect the front temperatures despite a slight increase in the front velocities. Further, as far as their swelling behavior is considered, all the synthesized hydrogels showed thermoresponsive features, with lower critical solution temperatures between 28 and 30 °C. Nevertheless, the swelling ratio of the covalently crosslinked systems was much lower than that of their supramolecular-crosslinked counterparts, with the latter exhibiting swelling degrees characteristic of superabsorbent hydrogel networks.

Feng and co-workers [47] succeeded in enhancing the mechanical properties of temperature-sensitive poly(*N*-isopropylacrylamide) hydrogels through the incorporation of organo-modified montmorillonite at different loadings. The fast front propagation efficiently inhibited phase separation phenomena, hence providing a homogeneous distribution of the nanoclay within the polymer matrix, resulting in a partially exfoliated structure. Further, the compressive strength of the hydrogel filled with 5 wt.% of clay increased by about 70% compared to that of the unfilled counterpart; moreover, this composite hydrogel showed the fastest temperature response among all the prepared composite hydrogels. Conversely, the nanoclay, irrespective of its loading, did not modify the lower critical solution temperature, which was around 33 °C. Finally, at a constant nanoclay loading (2 wt.%), both front temperature and velocity were found to increase when increasing the reactor tube diameter because of decreased heat loss via exchange with the environment; however, at the same time, the compressive strength slightly decreased.

Rassu and co-workers [48] demonstrated the suitability of frontal polymerization for preparing hydrogels made of methyl cellulose and poly(acrylamide), using *N*-*N*′-methylene-bis-acrylamide as a crosslinker at different concentrations (ranging from 0.1 to 2.0 mol%). To this end, the reaction was carried out in water and glycerol: the latter led to higher front temperatures and front velocities with respect to the counterparts synthesized in H_2_O due to the evaporation of the solvent and subsequent heat loss. Further, with a constant methyl cellulose content, progressively increasing the crosslinker amount led to a noticeable decrease in the swelling ratio and a remarkable improvement in mechanical behavior irrespective of the employed solvent.

**Figure 9:**
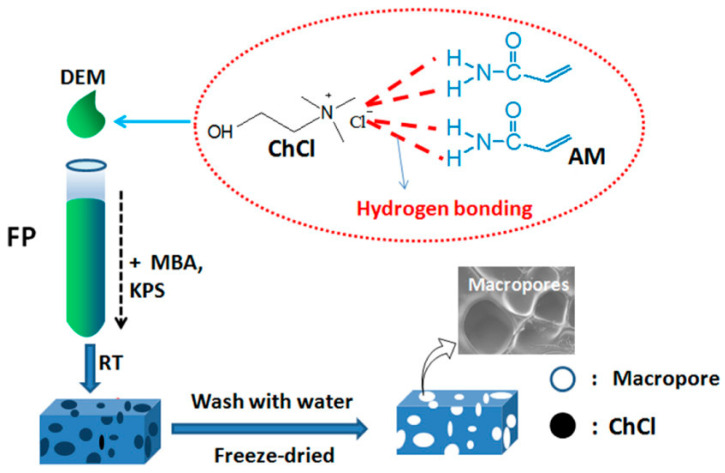
Scheme of the synthesis of macroporous polyacrylamide hydrogels obtained via the FP of deep eutectic monomers. Legend: AM = acrylamide; ChCl = choline chloride; DEM = deep eutectic monomer; FP= frontal polymerization; KPS = potassium persulfate; RT = room temperature. Reprinted with permission from [51]. Copyright American Chemical Society, 2020.

Another interesting approach that can be successfully exploited for preparing hydrogels for biomedical applications is the frontal polymerization of polymerizable deep eutectic monomers, i.e., a recently developed type of deep eutectic solvent that can be obtained by mixing quaternary ammonium and hydrogen bond donor monomers and is characterized by high thermal stability, low vapor pressure, low cost, high viscosity, and low toxicity [49,50]. In this context, Jiang and co-workers [51] synthesized macroporous polyacrylamide hydrogels via the frontal polymerization of deep eutectic monomers obtained by mixing several molar ratios of acrylamide and choline chloride, as schematized in Figure 9.

Increasing the molar ratio of acrylamide to choline chloride led to an increase in both front temperatures and front velocities. Further, the presence of choline chloride was beneficial for enhancing the conversion of acrylamide. As assessed via SEM analyses, due to the easy removal of the water-soluble choline chloride, the synthesized hydrogels exhibited a macroporous structure, which accounted for superfast responsive swelling and deswelling behavior when the hydrogels were alternately treated in acetone and in water.

### 4.2. Drug Delivery

The first hydrogel designed for drug delivery prepared via frontal polymerization was synthesized by Mariani’s group in 2009 [52]. Recently, Wang and co-workers [53] frontally polymerized a mixture containing *N*,*N*′-methylene-bis-acrylamide, acrylic acid (used as a functional monomer), *N*-isopropylacrylamide, and gatifloxacin (used as the template molecule) for preparing pH/temperature-sensitive hydrogel-based molecularly imprinted systems suitable for drug delivery purposes. Notably, these molecularly imprinted systems showed stimuli-sensitive recognition features toward target (template) molecules and could reversibly swell and shrink according to specific environmental changes (like temperature and pH). The imprinting efficiency was strictly dependent on both the acrylic acid/gatifloxacin ratio and *N*-isopropylacrylamide content. Moreover, compared to the counterparts prepared via classical batch polymerization, the frontally polymerized hydrogels showed higher imprinting effects in terms of their imprinting factor.

Feng et al. [54] exploited the frontal polymerization technique for preparing semi-interpenetrating polymer networks made of poly(*N*-isopropylacrylamide) and polyvinylpyrrolidone. The presence of an increasing polyvinylpyrrolidone loading induced shortened response times without altering the lower critical solution temperature of the poly(N-isopropylacrylamide). Furthermore, both the front temperatures and front velocities decreased when increasing the polyvinylpyrrolidone content because of the increase in the viscosity of the reaction system, which lowered buoyancy-driven convection, hence decreasing the heat transfer rate. Conversely, mechanical behavior, in particular the compressive strength of the obtained hydrogels, improved when increasing the polyvinylpyrrolidone loading, rising from 26.4 (for the poly(*N*-isopropylacrylamide) hydrogel) to 53.6 kPa (for the semi-interpenetrating hydrogel containing 20 wt.% of polyvinylpyrrolidone). Further, using aspirin as a model drug, it was possible to assess the suitability of the designed systems for drug delivery. In particular, it was found that not only was their drug loading capacity increased from 245 ((poly(*N*-isopropylacrylamide) hydrogel) to 422 mg/g (hydrogel containing 20 wt.% of polyvinylpyrrolidone) but also that drug release was more controllable in the presence of the semi-interpenetrating networks.

Then, the possibility of synthesizing semi-interpenetrating poly(*N*-isopropylacrylamide) hydrogels containing methylcellulose via frontal polymerization was demonstrated by Mariani and co-workers [55]. In particular, the effect of increasing amounts of *N*,*N*′ methylene-bis-acrylamide (employed as crosslinker within 0.1 and 2.0 mol%) on mechanical and swelling behavior (in two solvents, namely, water and dimethyl sulfoxide, which have different polarities) was thoroughly evaluated, elucidating the role of the methylcellulose at a constant loading (15 wt.%). Despite the negligible impact of the crosslinker on front temperatures and velocities, it remarkably affected the Young’s moduli of the hydrogels, regardless of the employed swelling solvent. Further, the lower critical solution temperature of the synthesized hydrogels (which exhibited the highest swelling ratio values at around 15 °C) was below 30 °C, and this was due to the incorporation of methylcellulose. Finally, the significant decrease (by two orders of magnitude) in the swelling ratios measured at room temperature (about 37,200%) and body temperature (around 240%) clearly confirmed the potential of these hydrogels for drug delivery purposes. 

**Figure 10:**
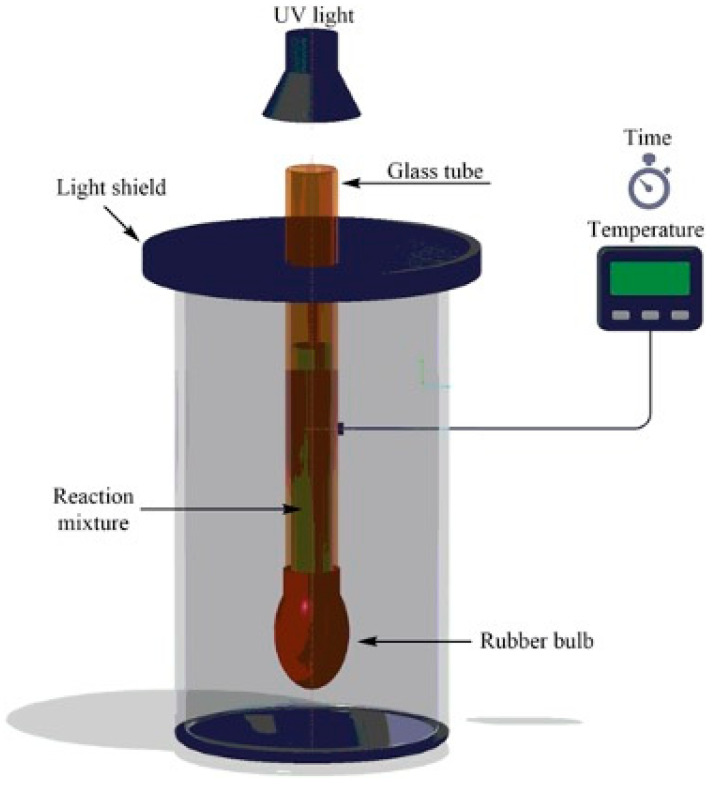
The device employed for frontal photopolymerization. Reprinted with permission from [56]. Copyright Wiley, 2019.

**Figure 11:**
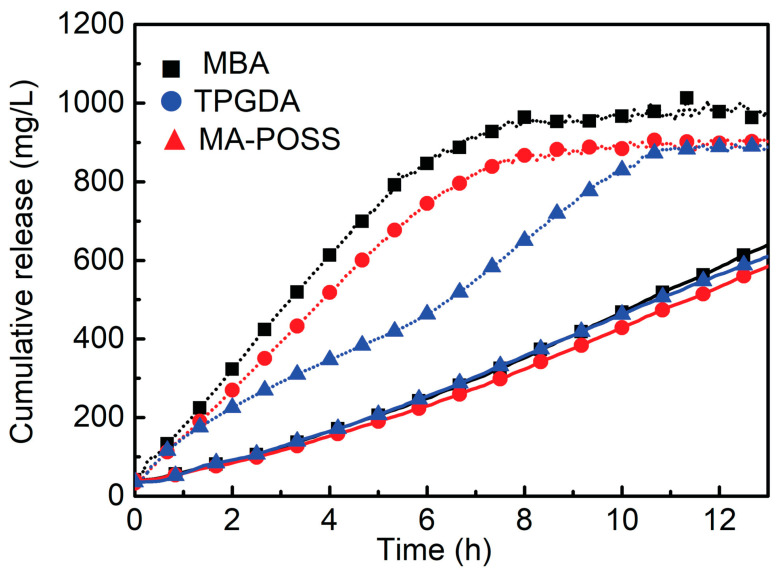
Drug release behaviors of poly(*N*-isopropylacrylamide) hydrogels containing different crosslinkers at 37 °C (solid line) and 20 °C (dotted line). Legend: MBA = *N*,*N*′-methylene-bis-acrylamide, used at 2 mol% loading; TPGDA = tri(propylene glycol) diacrylate at 2 mol% loading; MA-POSS = acrylopropyl polyhedral oligomeric silsesquioxane cage mixture at 0.5 mol% loading. Reprinted with permission from [56]. Copyright Wiley, 2019.

An interesting alternative to standard frontal polymerization for the obtainment of thermoresponsive poly(*N*-isopropylacrylamide) hydrogels suitable for drug delivery was quite recently proposed by Su and co-workers [56]. The use of UV radiation allows for operation at low pressure, reducing the temperature of the propagation front to a restrained level, hence avoiding possible risks of explosion and morphological defects in the obtained hydrogels because of partial monomer evaporation [57]. More specifically, the authors discussed the effects of the adopted experimental conditions (i.e., the types and concentrations of photo-initiators, namely, 2,4,6-trimethylbenzoyldiphenyl phosphine oxide or phenyl bis(2,4,6-trimethylbenzoyl)-phosphine oxide, and crosslinkers, namely, *N*,*N*′-methylene-bis-acrylamide, tri(propylene glycol) diacrylate; an acrylopropyl polyhedral oligomeric silsesquioxane cage mixture; and radiation intensity) on the frontal polymerization reaction and the overall properties of the obtained hydrogels. The general scheme of the device employed for the frontal photopolymerization is displayed in Figure 10. As in a typical preparation, the monomer, the photo-initiator, and the selected crosslinking agent were ultrasonicated in dimethylsulfoxide. Then, the resulting solution was poured into a glass tube encapsulated with a rubber bulb at the bottom to compensate for polymerization shrinkage. The filled tube was irradiated for 15 min with a medium-pressure mercury lamp, and the obtained hydrogels were immersed in deionized water for 24 h. Among the selected experimental parameters, only the radiation intensity turned out to increase both front temperatures and front velocities. Further, using aspirin as a model drug allowed for the assessment of the drug delivery behavior of the synthesized hydrogels as a function of the type of crosslinker. In particular, a significant increase in the drug release rates was first observed, regardless of the type of crosslinker employed. Then, after 10 h, an asymptotic plateau was achieved when the temperature was set below the lower critical solution temperature of poly(*N*-isopropylacrylamide) (Figure 11). Finally, because of its highly microporous structure, the hydrogel crosslinked with *N*,*N*′-methylene-bis-acrylamide showed a higher drug release rate compared to the counterparts crosslinked with the other crosslinkers.

**Figure 12:**
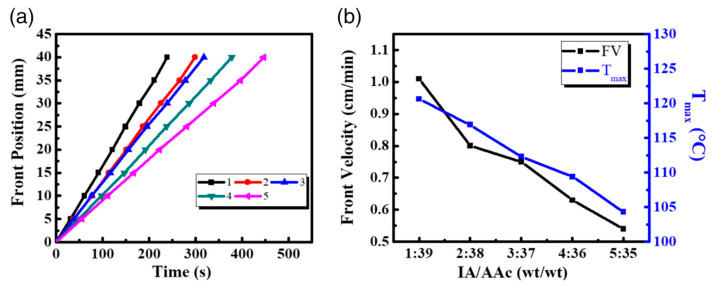
(**a**) Front position versus time for poly(itaconic acid-co-acrylic acid-co-acrylamide) hydrogels developed via FP at different IA/AAc weight ratios of (1) 1:39, (2) 2:38, (3) 3:37, (4) 4:36, and (5) 5:35 wt/wt (**b**) Frontal velocity (FV) and frontal temperature (*T*_max_) as a function of the itaconic acid/acrylic acid (IA/AAc) weight ratios at 10 wt.% acrylamide, 50 wt.%, glycerol, 0.24 wt.% *N*,*N*-methylene-bis-acrylamide, 0.1 wt.% ammonium persulfate, and 10 μL *N*,*N*,*N*′,*N*′-tetramethylethylenediamine. Reprinted with permission from [58]. Copyright Wiley, 2019.

Irfan and co-workers [58] exploited frontal polymerization for synthesizing a series of poly(itaconic acid-co-acrylic acid-co-acrylamide) hydrogels suitable for drug delivery purposes. More specifically, the effects of the itaconic acid/acrylic acid weight ratio (changing from 1:39 to 5:35 wt/wt) on the front temperatures and velocities were thoroughly investigated. As shown in Figure 12, the increase in this weight ratio accounted for a significant decrease in both frontal parameters. This finding was attributed to the limited reactivity of the allylic hydrogens of itaconic acid, which restricts its propensity to homo- or co-polymerize.

Moreover, standard gravimetric analyses were exploited for assessing the effect of the presence of itaconic acid on the swelling behavior of the synthesized hydrogels in H_2_O, keeping the acrylamide content constant at 10 wt.%. In particular, the swelling ratio values at equilibrium were found to increase from about 2500 to 4400% when increasing the itaconic acid/acrylic acid weight ratio from 1:39 to 3:37 wt/wt, respectively. Beyond this latter ratio, the swelling capability of the hydrogels significantly lowered because of the more difficult ionization of the polyelectrolyte, which, in turn, led to a tighter and denser hydrogel structure less prone to swell in water. 

**Figure 13:**
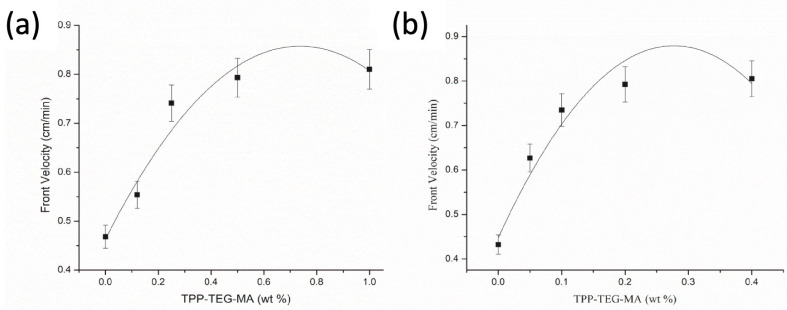
Front velocity vs. 5-(2-(2-(2-(2-phenoxyethoxy)ethoxy) ethoxy)-10,15,20-triphenylporphyrin methacrylate (TPP-TEG-MA) comonomer concentration in the frontal polymerization of poly(ethylene glycol) methyl ether acrylate (**a**) and di(ethylene glycol) methyl ether methacrylate (**b**) series. Reprinted with permission from [59]. Copyright Elsevier, 2022.

Quite recently, Martinez-Serrano and co-workers [59] succeeded in obtaining fluorescent hydrogels based on poly(ethylene glycol) methyl ether acrylate or di(ethylene glycol) methyl ether methacrylate and containing 5-(2-(2-(2-(2-phenoxyethoxy)ethoxy) ethoxy)-10,15,20-triphenylporphyrin methacrylate (which was purposefully synthesized). First of all, the incorporation of the porphyrin units was found to increase the glass transition temperature of the di(ethylene glycol) methyl ether methacrylate-based hydrogels only from −61 °C (for the hydrogel not containing the porphyrin methacrylate monomer) to about −15 °C. This finding was ascribed to the large volume of the porphyrin units with respect to the di(ethylene glycol) methyl ether methacrylate, which made the final hydrogels stiffer. Further, the front velocities showed an increasing trend when increasing the 5-(2-(2-(2-(2-phenoxyethoxy)ethoxy) ethoxy)-10,15,20-triphenylporphyrin methacrylate content (Figure 13). However, beyond 1 and 0.4 wt.% concentrations of the porphyrin methacrylate monomer (for the hydrogels based on poly(ethyleneglycol) methyletheracrylate and diethyleneglycol methylethermethacrylate, respectively), the steric effects exerted by the fluorescent monomer did not allow for the self-sustainment of the propagating front. Moreover, the increasing content of porphyrin units did not substantially affect the front temperatures.

**Figure 14:**
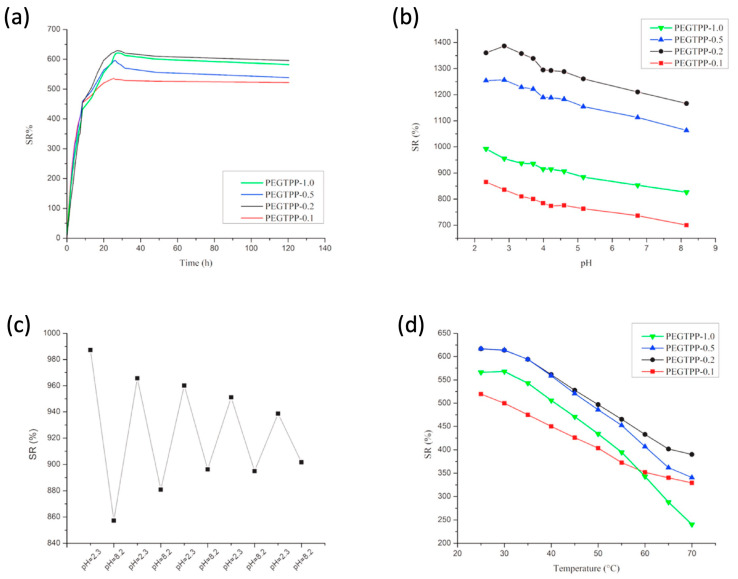
Swelling behavior of poly(ethyleneglycol)methylether acrylate (PEGTPP-X) series, where X represents the mol.% loading of the porphyrin methacrylate monomer: (**a**) swelling behavior at constant temperature, i.e., 25 °C, and pH = 7; (**b**) pH response; (**c**) pH reversibility response for PEGTPP-1.0, for which measurements were taken after equilibrium was reached; (**d**) temperature response. Reprinted with permission from [59]. Copyright Elsevier, 2022.

**Figure 15:**
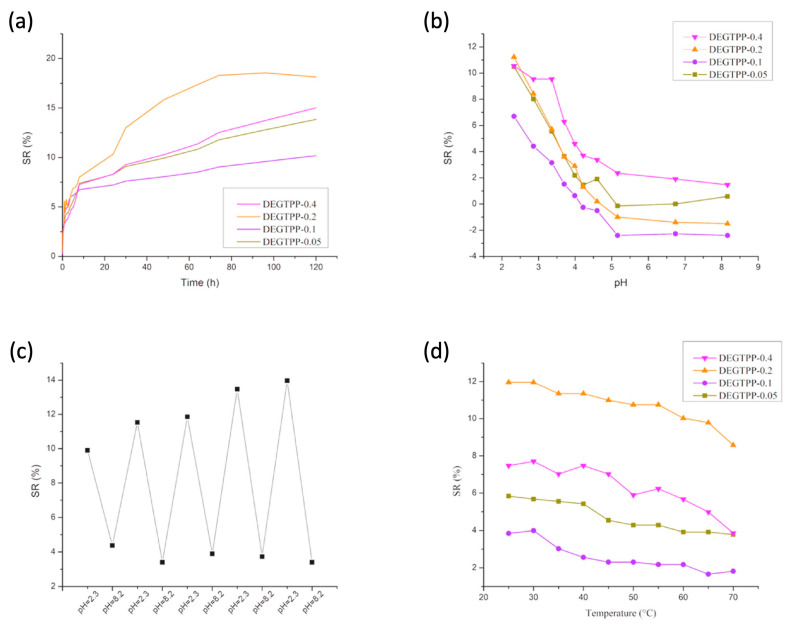
Swelling behavior of di(ethylene glycol) methyl ether methacrylate (DEGTPP-X) series, where X represents the mol.% loading of the porphyrin methacrylate monomer: (**a**) swelling behavior at constant temperature, 25 °C, and pH = 7; (**b**) pH response; (**c**) pH reversibility response for DEGTPP-0.4, for which measurements were taken after equilibrium was reached; (**d**) temperature response. Reprinted with permission from [59]. Copyright Elsevier, 2022.

Finally, as displayed in Figure 14 (for the poly(ethyleneglycol methylether acrylate series) and Figure 15 (for the diethyleneglycol methylether methacrylate series), the prepared hydrogels were both pH- and temperature-dependent. Indeed, both types of hydrogels showed reduced equilibrium swelling ratios when increasing the testing temperatures as well as when increasing the pH of the water medium. Moreover, diethyleneglycol methylether methacrylate exhibited swelling reversibility during immersion cycles in alternated low (2.3) and high (8.2) pH values (Figure 15c), unlike the systems derived from poly(ethyleneglycol) methylether acrylate (Figure 14c), which showed a loss of reversibility.

Very recently, Li and co-workers [60] exploited the frontal polymerization method for preparing pH-responsive and β-cyclodextrin-containing poly(acrylic acid-co-acrylamide) hydrogels. Toward this aim, a deep eutectic solvent mixture made of betaine (serving as a hydrogen bond acceptor), acrylic acid, and acrylamide (both serving as hydrogen bond donors) was employed. The acrylamide/acrylic acid/betaine molar ratio was set at 2:2:1; *N*,*N*-methylene-bis-acrylamide and potassium persulfate were utilized as a crosslinker and an initiator, respectively. Different β-cyclodextrin loadings were employed, namely, 0.25, 0.50, and 1.0 wt.%. Under the adopted experimental conditions, less than 6 min was enough to perform the frontal polymerization reactions. However, the presence of β-cyclodextrins led to a decrease in the polymerization rates, front velocities, and front temperatures. Conversely, both the crosslinking density and the number of H-bonds in the hydrogels increased when increasing the amount of β-cyclodextrins, hence leading to enhanced overall mechanical behavior. Further, as assessed via drug loading and release tests carried out with tetracycline hydrochloride (employed as a drug model), the incorporation of β-cyclodextrins accounted for a gradual increase in the drug loading and a gradual lowering in drug release due to the formation of host–guest inclusion complexes.

### 4.3. Self-Healing

The possibility of designing materials able to repair themselves after damage and recover (at least) their functionality utilizing the resources intrinsically available to them is very intriguing and has significantly motivated both the academic and industrial world over the last 20–25 years [61,62]. In this context, the past decade has witnessed the design, preparation, and application of self-healing, injectable hydrogels, for which self-healing relies on reversible chemical approaches. These advanced materials are capable of provisionally fluidizing when subjected to sufficient shear stresses and successively regaining their original mechanical features [63,64]. In further research efforts, the possibility of preparing frontally polymerized self-healing hydrogels has been thoroughly investigated: the next paragraphs summarize the most recent successful examples.

**Figure 16:**
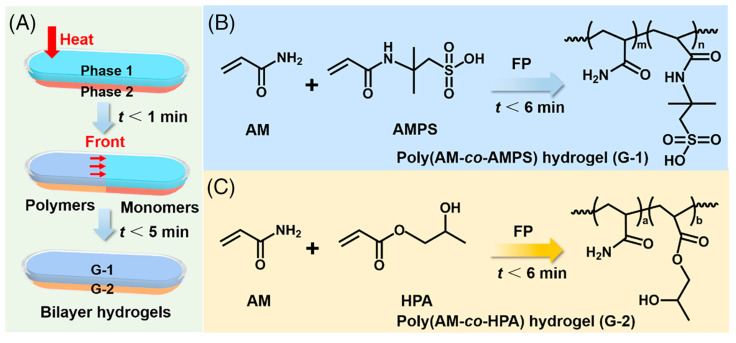
(**A**) Schematic illustrations of the synthesis of bi-layered hydrogels via horizontal bi-phase frontal polymerization. Rapid synchronized preparation of (**B**) poly(acrylamide-co-2-acrylamide-2-methylpropanesulfonic acid) (poly(AM-co-AMPS)) (G-1) and (**C**) poly(acrylamide-co-hydroxypropyl acrylate) (poly(AM-co-HPA)) (G-2) to form bilayer hydrogels. Reprinted with permission from [65]. Copyright Wiley, 2022.

Yang and co-workers [65] succeeded in obtaining frontally polymerized (in horizontal configuration) bi-layered anisotropic hydrogels made of poly(acrylamide-co-2-acrylamide-2-methylpropanesulfonic acid) and poly(acrylamide-co-hydroxypropyl acrylate). For the former layer, the weight ratio between acrylamide and 2-acrylamide-2-methylpropanesulfonic acid was set to 4:1, whereas a 1:1 acrylamide/hydroxypropyl acrylate weight ratio was chosen for the latter. *N*,*N*,*N*′,*N*′-tetramethylethylenediamine was employed as a crosslinker. As presented in Figure 16, the bi-layered structure was obtained by pouring the solution of poly(acrylamide-co-hydroxypropyl acrylate) into a horizontal glass tube and then adding the solution of poly(acrylamide-co-2-acrylamide-2-methylpropanesulfonic acid) as the second layer. Subsequently, to trigger the reaction and the propagation fronts, the left side of the glass tube was heated by means of a soldering iron for 20–30 s.

Both front temperatures and velocities were found to decrease when decreasing the weight ratio between the solutions of poly(acrylamide-co-2-acrylamide-2-methylpropanesulfonic acid) and poly(acrylamide-co-hydroxypropyl acrylate). This finding was ascribed to the higher heat loss in the solution of poly(acrylamide-co-2-acrylamide-2-methylpropanesulfonic acid) due to its direct exposure to the atmosphere.

**Figure 17:**
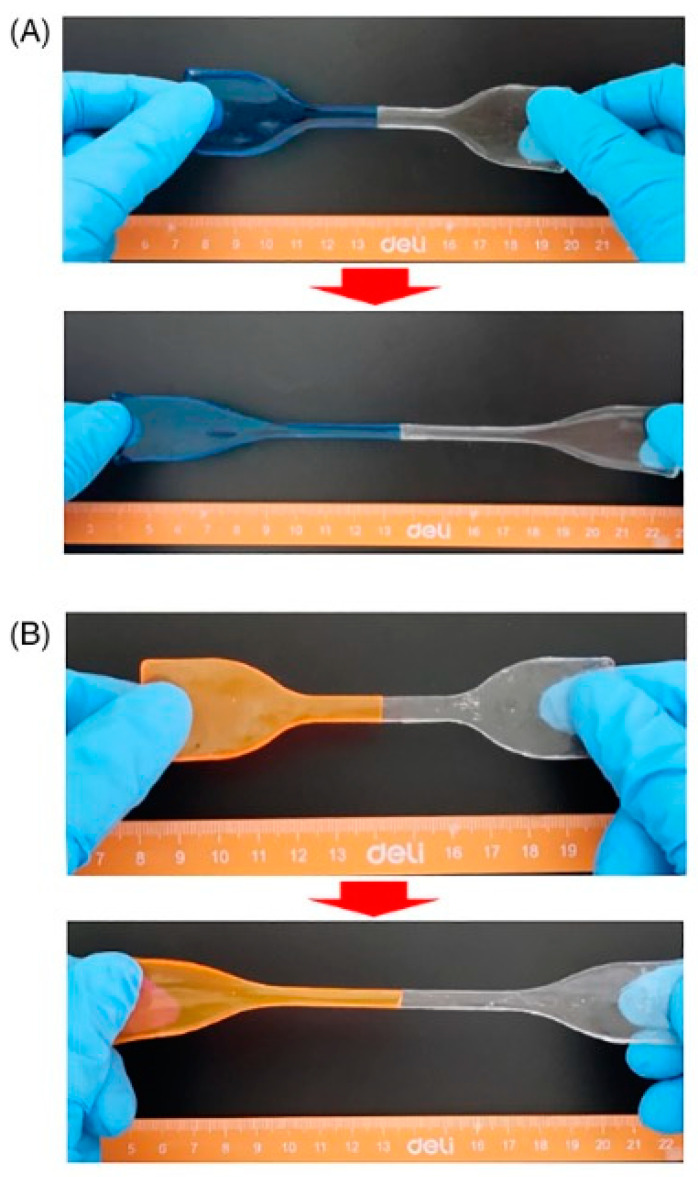
Digital photographs of self-healed samples of (**A**) poly(acrylamide-co-2-acrylamide-2-methylpropanesulfonic acid) and (**B**) poly(acrylamide-co-hydroxypropyl acrylate) hydrogels obtained by slicing the bi-layered system (prepared with 5:5 weight ratio between the two polymer solutions). Each sample was cut into two separate halves, one of which was dyed; then, the two halves were put together for 24 h at room temperature and finally merged into a complete sample capable of sustaining intense stretching. Reprinted with permission from [65]. Copyright Wiley, 2022.

All the bi-layered hydrogels showed high hydrophilicity and excellent water absorption ability. Among the different investigated weight ratios between the two polymer solutions, that of 5:5 exhibited the highest equilibrium swelling ratio in water (around 3370%) at a neutral pH. Moreover, as assessed through tensile tests, the cut surfaces of the hydrogels showed self-healing abilities, even without the use of any external stimuli; the self-healed systems were able to withstand robust stretching (Figure 17).

Finally, the bi-layered hydrogels possessed good cytocompatibility and low cytotoxicity, with 78.13% cell survival, as demonstrated by means of in vitro cell culture tests analyzing L929 fibroblasts.

**Figure 18:**
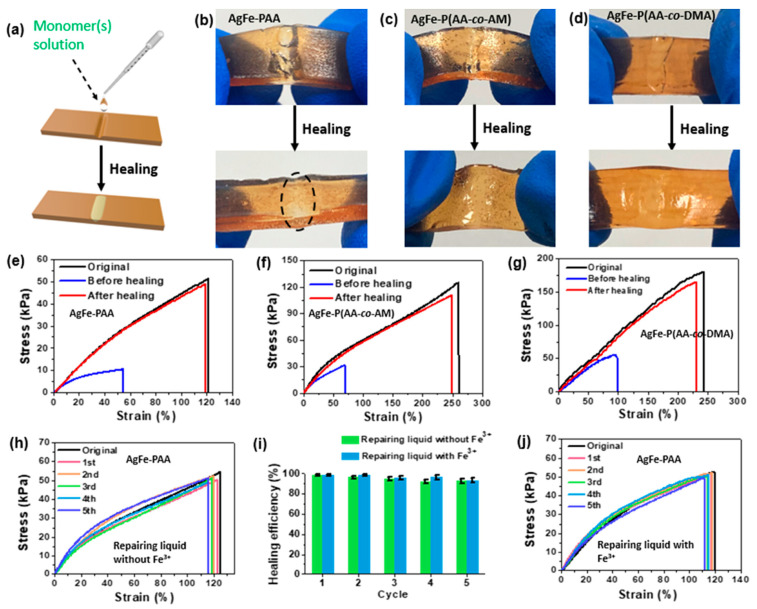
(**a**) Schematic illustration of the interfacial-ignited-gelation-promoted healing of a covalently cross-linked polymer hydrogel. (**b**–**d**) Pictures showing the interfacial ignited gelation-healing of the covalently cross-linked Ag^+^/Fe^3+^-poly(acrylic acid) (AgFe-PAA; polymer concentration: 20 wt.%), Ag^+^/Fe^3+^-poly(acrylic acid-co-acrylamide) (AgFe-P(AA-*co*-AM); polymer concentration: 20 wt.%; 2:1 acrylic acid/acrylamide mole ratio), and Ag^+^/Fe^3+^-poly(acrylic acid-co-*N*,*N*-dimethylacrylamide) (AgFe-P(AA-*co*-DMA; polymer concentration: 20 wt.%; 2:1 acrylic acid/ *N*,*N*-dimethylacrylamide mole ratio) hydrogels. (**e**–**g**) Stress–strain curves illustrating the interfacial-ignited-gelation-promoted healing of different hydrogels in panels (**b**–**d**). (**h**,**j**) Stress–strain curves showing the repeated interfacial ignited gelation-healing of the AgFe-PAA hydrogels using “repairing liquids” without and with Fe^3+^. (**i**) Healing efficiencies of the hydrogels in (**h**,**j**) at different healing cycles (up to 5 cycles). Reprinted with permission from [66]. Copyright American Chemical Society, 2023.

Liu and co-workers [66] exploited an interfacial ignited gelation process in order to obtain Ag^+^/Fe^3+^-poly(acrylic acid), Ag^+^/Fe^3+^-poly(acrylic acid-co-*N*,*N*-dimethylacrylamide), and Ag^+^/Fe^3+^-poly(acrylic acid-co-acrylamide) self-healing hydrogels. The reaction occurred in water and was induced by means of a frontal polymerization process. Working at room temperature in an air atmosphere and simply utilizing the acrylic acid or acrylic acid/acrylamide aqueous solutions as the repairing medium led to the occurrence of an interfacial ignited gelation process that repaired the two types of synthesized hydrogels. Healing efficiencies beyond 90%, even after five repeated healing cycles, were observed (Figure 18). In particular, high healing efficiencies and heating times as low as 1 min were observed for the hydrogels with a high polymer content (i.e., 92 wt.%).

Recently, Li et al. [67] demonstrated the suitability of frontally polymerized deep eutectic monomers for obtaining poly(urea-co-acrylamide-co-choline chloride) hydrogels containing sodium alginate as a filler (from 0.5 to 8.0 wt.% loading). The effects of sodium alginate on the front velocities and temperatures as well as the mechanical, swelling, and self-healing features of the resulting hydrogels were thoroughly investigated. More specifically, because of the decrease in the number of acrylamide units per unit volume and, therefore, in the heat generation per unit time, both the front velocities (1.23 vs. 0.66 cm/min for the unfilled hydrogel and the counterpart containing 8 wt.% of filler, respectively) and temperatures (from about 136 to 100 °C, respectively) lowered when increasing the sodium alginate content. Conversely, the presence of increasing filler loadings accounted for an increase in the maximum tensile strength of the hydrogels (from 56.4 to 163.3 kPa for the unfilled hydrogel and the counterpart containing 8 wt.% of filler, respectively). A similar trend was also observed regarding the swelling ratios and self-healing efficiencies: for the latter, a value as high as 94.4% was achieved after 48 h for the hydrogels containing the highest loading of sodium alginate. Replacing sodium alginate with ZnO nanoparticles (in the range 0.4–1.2 wt.%) endowed the obtained hydrogels with interesting antibacterial features toward both *E. coli* and *S. aureus* [68]. More specifically, the inhibition rate progressively increased when increasing the nanofiller content, reaching values beyond 80% for the highest loading.

**Figure 19:**
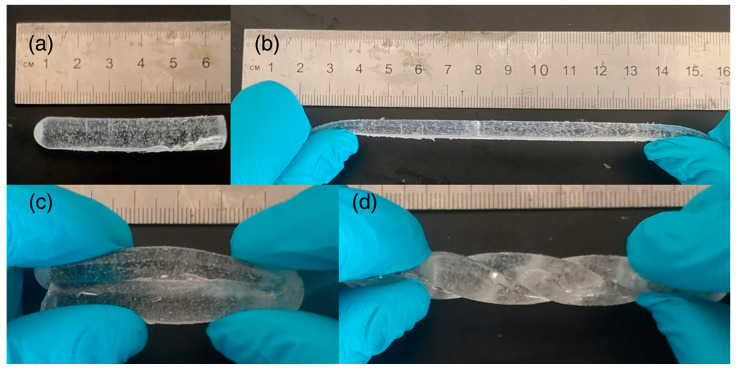
Hydrogel (2:1:1 acrylamide/choline chloride/glycerol molar ratio): (**a**) image showing the hydrogel’s original length; (**b**) images showing how the hydrogel was able to easily stretch to more than twice its original length, (**c**) be folded at will, and (**d**) remain twistable after folding without damage. Reprinted with permission from [69]. Copyright Wiley, 2023.

**Figure 20:**
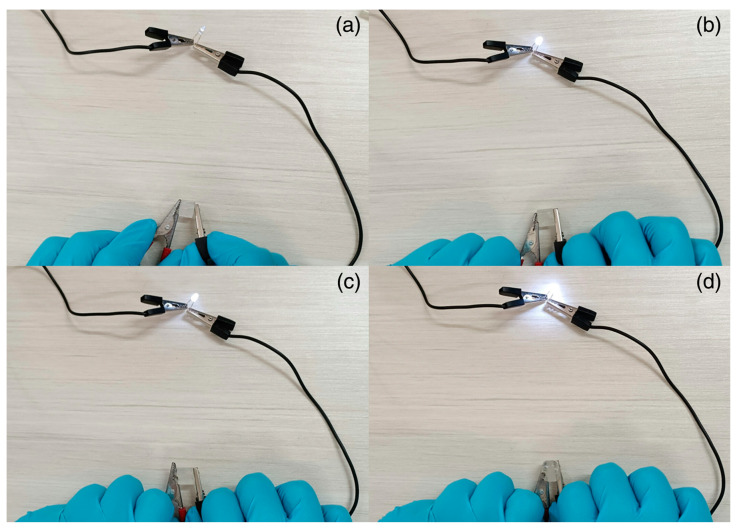
Photographs of LED light brightness after connecting different hydrogel samples: (**a**,**b**) hydrogel (1:1:1 acrylamide/choline chloride/glycerol molar ratio) before and after water absorption; (**c**,**d**) the same hydrogel before and after compression. Reprinted with permission from [69]. Copyright Wiley, 2023.

Pursuing this line of research, the same group [69] exploited a deep eutectic monomer mixture made up of different molar ratios of acrylamide, choline chloride, and glycerol to obtain multifunctional frontally polymerized hydrogels. *N*,*N*-methylene-bis-acrylamide and potassium persulfate were used as a crosslinker and an initiator, respectively. Increasing the acrylamide content in the hydrogel formulation led to increased front temperatures and velocities. Further, because of the formation of hydrogen bonding interactions taking place between acrylamide and choline chloride/glycerol, the resulting hydrogels showed very good mechanical features. High stretchability (with around 350% maximum elongation) and twistability (Figure 19) and a good self-healing capacity were observed. Finally, the presence of increasing amounts of glycerol in the deep eutectic monomer mixture induced an augmented ionic conductivity of the hydrogels (Figure 20), making them potentially suitable as compression sensors.

**Figure 21:**
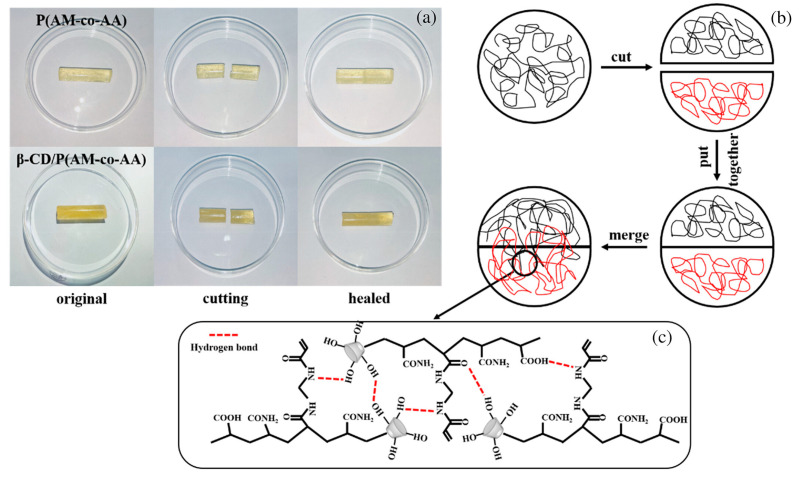
(**a**) Self-healing ability of the hydrogel without (P(AM-co-AA)) and with 2.0 wt.% of β-cyclodextrins (β-CD/P(AM-co-AA)); concerning the former, an obvious dividing line at the cross-section appears, while for the latter, this line is almost invisible at the cross-section. (**b**) Scheme of the self-healing process of β-CD/P(AM-co-AA) hydrogels, which occurs through hydrogen bonding. (**c**) β-CD/P(AM-co-AA) hydrogen bonds formed between the molecular chains of the hydrogel. Reprinted with permission from [70]. Copyright Wiley, 2023.

In a further research effort [70], a deep eutectic monomer mixture consisting of choline chloride, acrylamide, and acrylic acid (in a 1:1:1 molar ratio) was employed for synthesizing frontally polymerized composite hydrogels containing β-cyclodextrins (from 0.25 to 2.0 wt.%). *N*,*N*-methylene bisacrylamide and potassium persulfate were employed as a crosslinker and an initiator, respectively. Increasing the content of β-cyclodextrins induced an increase in H-bond formation, hence improving the overall mechanical behavior of the hydrogels. In particular, compressive and tensile strength were increased 2.26- and 4.12-fold in comparison to the hydrogels’ unfilled counterparts. Moreover, the presence of β-cyclodextrins provided the hydrogels with interesting self-healing properties, with an efficiency approaching 92% for the systems containing the highest filler loading after 48 h (Figure 21).

**Figure 22:**
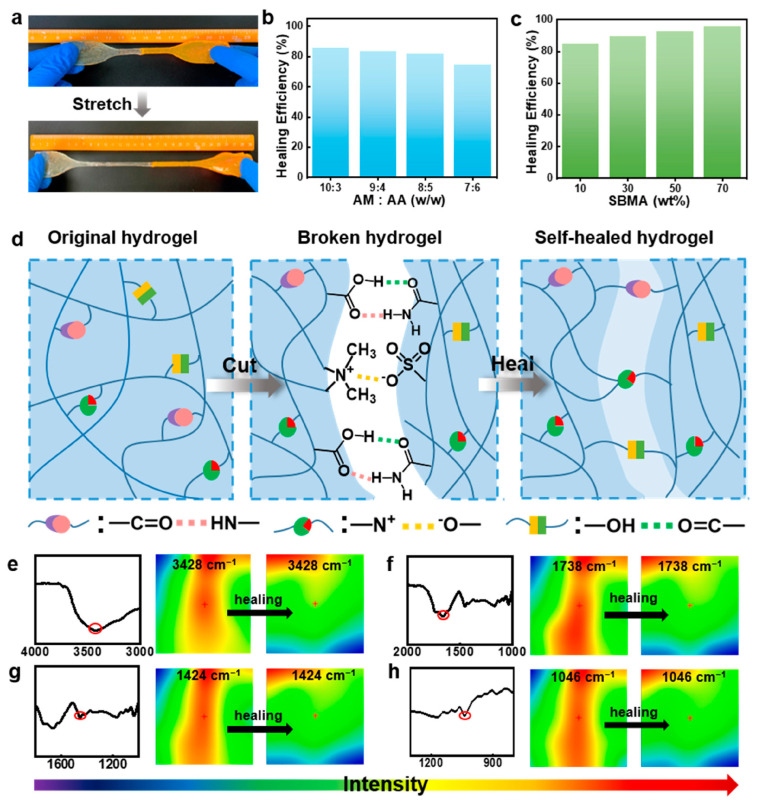
(**a**) Photographs of a healed sample of a poly(acrylamide-*co*-acrylic acid-*co*-3-[Dimethyl-[2-(2-methylprop-2-enoyloxy)ethyl]azaniumyl]propane-1-sulfonate) hydrogel being stretched. (**b**,**c**) Self-healing efficiency of the hydrogels prepared with (**b**) different acrylamide/acrylic acid (AM/AA) mass ratios (at 10 wt.% of 3-[Dimethyl-[2-(2-methylprop-2-enoyloxy)ethyl]azaniumyl]propane-1-sulfonate (SBMA)) and (**c**) different SBMA concentrations (AM/AA mass ratio: 8:5 after 24 h at room temperature. (**d**) Self-healing mechanism via ionic association and hydrogen bonding interactions shown in schematic. (**e**–**h**) FT-IR spectroscopy and infrared pictures of these hydrogels before and during self-healing. Red circles indicate the occurrence of hydrogen bonding and electrostatic interactions within the synthesized hydrogels. Reprinted from [71] under CC-BY 4.0 License.

Very recently [71], frontal polymerization was exploited for obtaining highly swellable, stretchable, and self-healing hydrogels through the copolymerization of acrylamide, 3-[dimethyl-[2-(2-methylprop-2-enoyloxy)ethyl]azaniumyl]propane-1-sulfonate, and acrylic acid. The obtained hydrogels exhibited superabsorbent features and were sensitive to pH: their swelling ratios were as high as 11,802% in water and 13,588% in an alkaline environment. Finally, these hydrogels showed high self-healing abilities, with a healing efficiency of up to 95% (Figure 22).

### 4.4. Other Applications: Electrically Conductive and Photothermic Hydrogels

The versatility and tunability of hydrogels prepared via frontal polymerization are so high that they recently have started to be employed for purposes other than those in the biomedical and pharmaceutical sectors. This paragraph will summarize the most recent outcomes.

**Figure 23:**
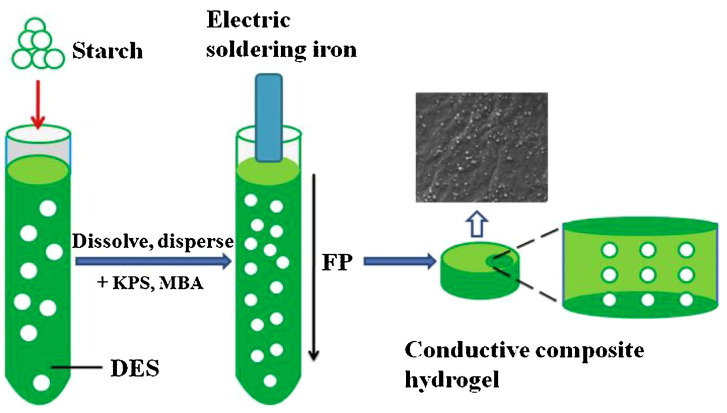
Scheme of the preparation of poly(ionic liquid)/starch hydrogels. Legend: DES = deep eutectic monomer mixture; FP = frontal polymerization; KPS = potassium persulfate; MBA = *N*,*N*-methylene-bis- acrylamide. Reprinted with permission from [72]. Copyright Elsevier, 2021.

As far as electrical conductivity (already mentioned in the previous paragraph as one of the possible features of frontally polymerized multifunctional hydrogels) is considered, Chen and co-workers [72] demonstrated the suitability of starch (containing 25 wt.% of amylose) for enhancing the ionic conductivity of frontally polymerized hydrogels. For this purpose, a deep eutectic monomer mixture made of acrylic acid, acrylamide, and choline chloride (1:1:1 molar ratio) was employed, using potassium persulfate and *N*,*N*-methylene-bis-acrylamide as an initiator and a crosslinker, respectively. The general scheme of the adopted synthetic procedure is shown in Figure 23.

**Figure 24:**
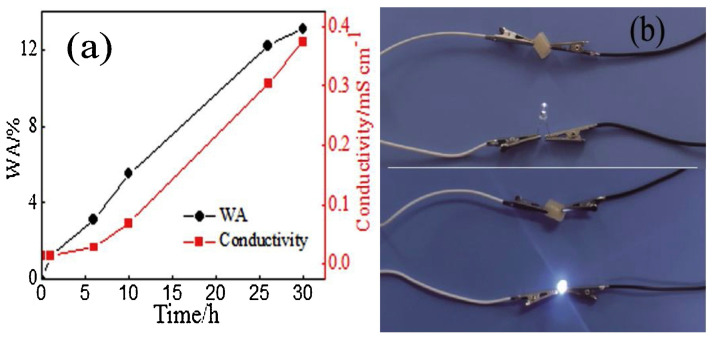
(**a**) Conductivity and water absorption (WA) vs. time for the hydrogel containing 10 wt.% of starch. (**b**) Digital photographs of the brightness of the same hydrogel connected to a LED bulb before and after water absorption. Reprinted with permission from [72]. Copyright Elsevier, 2021.

First of all, the presence of increasing starch loadings accounted for a decrease in the front velocities, while its effect on front temperatures was very limited. Further, thanks to the strong H-bond interactions with the deep eutectic monomer system, this biomacromolecule promoted an increase in the tensile and compressive strength of the hydrogels as well as in water absorption and electrical conductivity (Figure 24).

**Figure 25:**
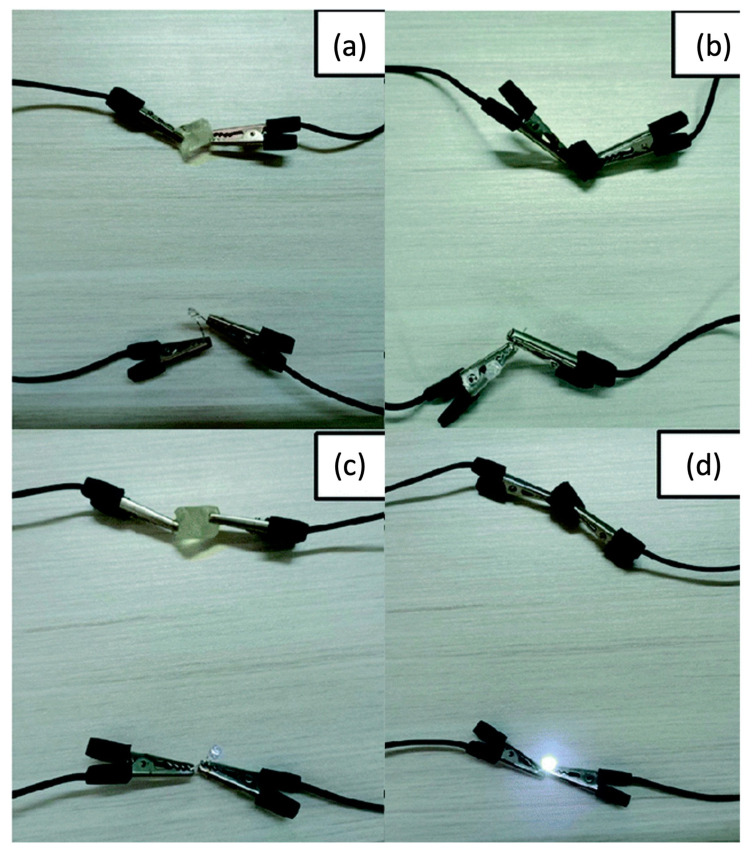
Brightness changes of LED connected to hydrogel circuit before and after absorbing water: (**a**) unfilled hydrogel before water absorption; (**b**) hydrogel containing 1.0 wt.% of N-doped carbon nanotubes before water absorption; (**c**) unfilled hydrogel after water absorption; (**d**) hydrogel containing 1.0 wt.% of N-doped carbon nanotubes after water absorption. Adapted from [73] under CC-BY 3.0 license.

A similar approach for obtaining electrically conductive hydrogels via frontal polymerization was employed by Li and co-workers [73], who incorporated N-doped carbon nanotubes (at a 0.2 to 1.0 wt.% loading) into a deep eutectic monomer mixture consisting of choline chloride, acrylic acid, and acrylamide (at a constant 1:1:1 molar ratio). Again, potassium persulfate and *N*,*N*-methylene-bis-acrylamide were employed as an initiator and a crosslinker, respectively. The presence of increasing amounts of the nanofiller accounted for a gradual increase in both front velocities and temperatures due to the viscosity increase in the reactive medium. Furthermore, because of the strong interfacial connection between the N-doped carbon nanotubes and the hydrogel networks, both tensile and compressive strength increased, presenting values of 5.42 and 4.29 MPa, respectively, which were about 4.7 and 2.1 times those obtained for the unfilled hydrogel. Finally, the conductivity of the hydrogels embedded with 1.0 wt.% of nanofiller was as high as 0.42 mS cm^−1^, i.e., about 4.2 times higher than that of the unfilled counterpart: a very bright light emission was observed for this nanocomposite hydrogel when connected to a LED bulb after water absorption (Figure 25).

**Figure 26:**
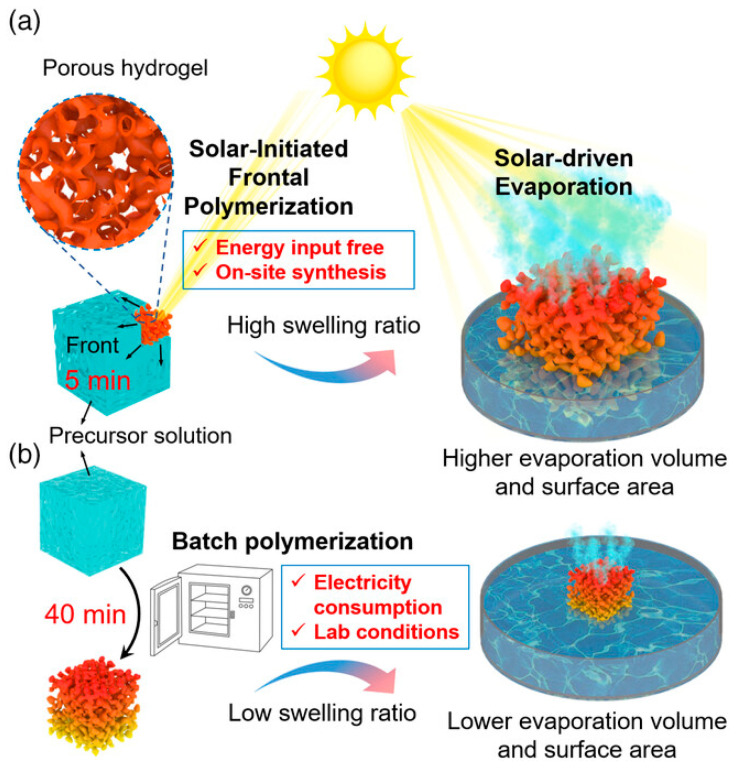
Schematic of solar-initiated frontal polymerization (**a**) and batch polymerization (**b**) for preparing hydrogel evaporator and evaporation processes. Reprinted with permission from [74]. Copyright Wiley, 2022.

Liang and co-workers [74] demonstrated the possibility of using solar-initiated frontal polymerization for obtaining poly(acrylamide-co-acrylic acid)/carbon black hydrogels suitable for photothermic seawater desalination. The filler loading was changed from 0.4 to 2.2 wt.%, while the acrylic acid/acrylamide weight ratio was kept constant at 3.7. The performance of the hydrogels obtained via frontal polymerization was compared with that of the same systems prepared by means of batch polymerization. A general scheme of the adopted strategy is presented in Figure 26. The combination of the peculiar honeycomb-like macroporous structure of the frontally polymerized hydrophilic hydrogels with the hydrophobicity of the carbon black clusters allowed for broadband light absorption and enhanced water transportation. In particular, the evaporation rate was as high as 2.42 kg m^−2^ h^−1^, which resulted in 92.8% light-to-vapor efficiency under one-sun irradiation. In addition, the solar evaporation flux was significantly boosted thanks to the significant swelling behavior of the FP-derived hydrogel evaporator, which was able to expand to 900 and 2700% of its original surface area and volume, respectively.

## 5. Conclusions and Perspectives

Frontal polymerization was invented in the 1970s [75] and, for a couple of decades, was considered an interesting example of phenomena related to nonlinear dynamics only [16]. Since the early years of this century, studies related to its feasibility when used alongside new monomeric systems, kinetics, and mechanisms have been added, almost to the point of replacing the originals entirely. By now, FP is a mature technique and, as such, is attracting the interest of an increasing number of research groups around the world. In particular, attention is now focused primarily on its practical applications, of which those involving hydrogels are among the most widely investigated.

As we have pointed out in previous paragraphs, FP allows for obtaining smart hydrogels, among which we would like to mention those exhibiting self-healing and stimulus-responsive (e.g., with respect to temperature and pH) characteristics.

Moreover, FP has been demonstrated to be a reliable and efficient approach for obtaining hydrogels in a short time and with limited energy consumption (indeed, external energy is only needed to trigger the reaction), leading to tailored and regular structures with enhanced thermo-mechanical, self-healing, and stimuli-responsive properties, among others. Compared to batch polymerization techniques, as clearly demonstrated in the pioneering research works, FP usually accounts for the higher conversion, higher polymerization rates, and improved thermal and mechanical features of the resulting polymerized or cured formulations [11,12,21]. Furthermore, in some cases, FP was found to be the only way to achieve particular characteristics (e.g., nanocomposite hydrogels characterized by highly homogeneously dispersed nanofillers). In fact, FP has been considered a fast method for obtaining new materials with the advantage of preventing any reaggregation/separation phenomena that may occur during monomer-to-polymer conversion, thus also making purification processes almost unnecessary. These peculiarities have stimulated the latest research works—as discussed in the present review—which focus on the FP technique only, without a comparison with the batch processes.

Although the use of the coupling between FP and hydrogels is still limited to a small number of systems, it may be easily foreseen that more interdisciplinary efforts will lead to a rapid increase in the variety of hydrogel recipes (i.e., monomers, oligomers, (nano)fillers, and additives), hence widening the potential exploitation of the final materials.

However, there are still aspects that limit the possibilities of their use. In terms of the technology readiness level, the maturity achieved by FP now requires further significant efforts to move from the level of technology validated in a relevant environment to subsequent levels that, through prototype demonstration in an operational environment, will allow for its practical use. From the point of view of research developments, it is still necessary to solve the heat dissipation issue. Toward this aim, catalytic or activating systems need to be developed; these systems allow for the propagation of fronts at relatively low temperatures and are chemically compatible with aqueous media. This will make it possible to not only operate in water but also to expand the number of exploitable monomers and, consequently, polymers. In addition, it will be possible to make polymeric materials with geometries that are not always accessible by all FP systems (e.g., thin films and fibers). This will also make it possible to focus more on the use of biobased chemicals even if they have relatively high molecular mass.

Further, more interaction with scientists having biological expertise will be required to design and prepare biocompatible systems of biomedical interest. It may even be speculated that these materials could be obtainable in situ within living organisms upon being triggered by an appropriate external stimulus.

Last but not least, frontal polymerization appears to be a natural candidate for implementation in 3D printing techniques used in biomedical applications, in which the generation of an artifact occurs as a result of the layer-by-layer deposition of monomers. In this setting, FP could be exploited for continuously polymerizing the deposited layers.

## Data Availability

Not applicable.
